# Population pharmacokinetics study of tacrolimus in liver transplant recipients: a comparison between patients with or without liver cancer before surgery

**DOI:** 10.3389/fphar.2024.1449535

**Published:** 2024-08-23

**Authors:** Haihong Bai, Juping Yun, Zihe Wang, Yingmin Ma, Wei Liu

**Affiliations:** ^1^ Department of Pharmacy, Beijing YouAn Hospital of Capital Medical University, Beijing, China; ^2^ Department of Respiratory and Critical Care Medicine, Beijing YouAn Hospital of Capital Medical University, Beijing, China

**Keywords:** tacrolimus, liver transplantation, population pharmacokinetics, liver cancer, therapeutic drug monitoring

## Abstract

**Background and Objective:**

The main challenge for immunosuppressive therapy using tacrolimus in liver transplantation is the considerable variability in its oral bioavailability and the narrow treatment range. Many population pharmacokinetic (PopPK) models have been established to precisely estimate the PK variability of tacrolimus in liver transplant recipients. However, it remains unclear whether there is a significant difference in the PK behavior of tacrolimus between patients with or without liver cancer before surgery. Therefore, we aimed to compare the differences of PK parameters and simulate exposures of tacrolimus between populations preoperatively diagnosed with liver cancer or not by PopPK modeling.

**Methods:**

In total, 802 blood concentrations of tacrolimus from 196 patients (118 liver cancer and 78 non-liver-cancer samples) were included in this study. Demographic data and clinical parameters were integrated to perform a PopPK analysis using the nonlinear mixed-effects modeling approach. Potential covariates were evaluated by using a stepwise method. Goodness-of-fit plot and bootstrap were performed to assess the model stability and predictive performance. Simulations were introduced to optimize dosing regimens of both the liver cancer and non-liver-cancer groups according to the guidance.

**Results:**

The PK of tacrolimus was best described by a one-compartment model with first-order absorption and linear elimination, with weight and direct bilirubin as the significant covariates. In the process of constructing the basic model, we tried to separately estimate the PK parameters in liver cancer and non-liver-cancer populations. The results showed that the PK parameters in the two populations were similar, and the individual variation in Ka in non-liver-cancer subjects was large. Hence, the final model did not distinguish between the two populations. Moreover, a minor increase of less than 10% was observed in the simulated exposure in the patients preoperatively diagnosed with liver cancer compared with that in non-liver-cancer groups.

**Conclusion:**

The established PopPK model was capable of optimizing tacrolimus dosing in whole populations who underwent liver transplantation. Although a minimal difference was found in tacrolimus exposure between the liver cancer and non-liver-cancer groups, more research is warranted to explore the differences between the two populations in the future, given the potential limitations of this study.

## 1 Introduction

Tacrolimus is a potent calcineurin inhibitor widely used for immunosuppressive regimens after liver transplantation. Lower rejection rates of allograft and thus improved clinical effectiveness were achieved in recent years due to the introduction of tacrolimus as a therapeutic drug (Boada-Pérez et al., 2024; Maslauskiene et al., 2024). However, the oral bioavailability of tacrolimus is highly variable, from 5% to 93%, and it also extensively binds to erythrocytes in whole blood (85%–95%) (Kirubakaran et al., 2020). Furthermore, the therapeutic window of tacrolimus is extremely narrow for immunosuppressive monotherapy, ranging from 5.0 to 15.0 ng/mL (Wang et al., 2023). These disadvantages pose significant challenges for maintaining the desired blood concentration of tacrolimus, and as such, patients may be at a great risk of acute rejection or drug-related toxicity.

Therapeutic drug monitoring (TDM) of whole-blood tacrolimus trough concentration is routinely adopted to guide the optimal dosage (Shimada et al., 2024; Vogeser and Habler, 2024). However, determining the individualized dosage of tacrolimus remains challenging. First, there are no previous TDM data for reference in the early post-transplantation phase (Chen et al., 2020). Additionally, the patients undergoing surgery often experience anemia, decreased albumin, organ dysfunction, and complex combination therapies, further exacerbating the variation in the *in vivo* pharmacokinetic (PK) behavior of tacrolimus (Xiao et al., 2024). Therefore, appropriate dose adjustment immediately after transplantation using the TDM method becomes more complicated.

Fortunately, population PK (PopPK) has become a popular approach for estimating the related PK parameters and determine significant covariates that impact PK variability of the target drugs (Abderahmene et al., 2024; Khamlek et al., 2024). By integrating Bayesian estimations, this technology facilitates more precise target concentration prediction and more expedited dosage adjustment, especially in situations where patients are clinically unstable or the drug concentrations have not reached a steady state. So far, many research studies have emphasized on tacrolimus treatment based on PopPK (Du et al., 2022; Teng et al., 2022). Depending on these developed models, several clinical determinants, such as body weight, hepatic function, hemoglobin, and hematocrit, have been explored and proven to deeply influence the PK characteristics of tacrolimus in patients after liver transplantation (Cai et al., 2022; Li et al., 2023; Ponthier et al., 2023). Therefore, clinicians could accurately guide tacrolimus dosing in liver transplant recipients rather than relying solely on personal experience.

Despite a promising prediction performance of the concentrations of tacrolimus after liver transplantation based on these published studies, there is still a limitation that needs to be fully elucidated. Liver cancer is one of the leading causes of cancer-related death in the world, and over 50% of the globally reported incidences and deaths are in China (Liu and Liu, 2022; Zhang et al., 2022). Compared to ablation and hepatectomy, liver transplantation could lead to a better prognosis, significantly reducing the risk of recurrence of liver cancer from 50%–60% to approximately 10% and improving the median survival to as high as 10 years (Mehta, 2024; Yankol et al., 2024). Therefore, with liver cancer accounts for nearly half of the total number of liver transplant cases in China, which is significantly higher than 5%–10% of liver transplant cases in European and American countries (Ju et al., 2023). However, to the best of our knowledge, no research pays special attention to elucidate the question that whether any PK variability of tacrolimus will be introduced due to the changes of the composition of the study populations. Since liver cancer is a systemic disease with abnormal molecular mutations, the metabolism of tacrolimus in these patients may be more susceptible to the clinical states compared to patients with other liver-related diseases (Gorji et al., 2023).

To address the above issue, the present research primarily aimed to 1) develop a PopPK model of tacrolimus in liver transplant recipients and compare the differences in PK parameters in patients with or without liver cancer before surgery; 2) identify the factors that affect the PK variability of tacrolimus; 3) simulate the exposure of tacrolimus in the two subgroups according to the guidance. Based on the results, we expect that this new study will find more application in the individual therapy of tacrolimus.

## 2 Materials and methods

### 2.1 Subjects and clinical data collection

A total of 196 patients who underwent the first liver transplantation in Beijing YouAn Hospital of Capital Medical University, from November 2021 to December 2023, were included in this study. The dosages and TDM records of tacrolimus were collected from the first day after surgery to the day of discharge from the hospital for each patient. Age, gender, weight, preoperative disease diagnosis, tacrolimus dose, time of tacrolimus administration, clinical data, and sampling time of each patient were collected retrospectively.

### 2.2 Drug administration

All the enrolled patients received an immunosuppressive regimen containing tacrolimus (Prograf, Astellas, Dublin, Ireland), mycophenolate mofetil, and methylprednisolone. The dosage of tacrolimus was empirically adjusted according to the tacrolimus trough concentrations and clinical indexes. Patients were instructed to take the drug on an empty stomach at a fixed time every morning (08:00) and evening (20:00) 6–48 h after liver transplantation.

### 2.3 Therapeutic drug monitoring

In the post-transplantation period, 4.0 mL of whole blood was first collected in EDTA•K2 pretreated tubes 0.5–1 h before morning administration or at the time required in clinical practice. Then, the obtained blood samples were stored at 4°C for further analysis. Finally, the concentrations of tacrolimus in whole blood were determined with a validated liquid chromatography–mass spectrometry (LC–MS)-based method in the predefined concentration range of 1.0–50.0 ng/mL.

### 2.4 PopPK analysis

The base model included a structural model and a random-effects model. The structural model examined the number of compartments, the mode of absorption, and the mode of elimination. The random-effects model included inter-individual variability (IIV) and residual unexplained variability (RUV). In addition, inter-occasion variability (IOV) was introduced if necessary. The differences between the PK parameters of liver cancer and non-liver-cancer populations were examined separately during the establishment of the base model.

For the covariate model, empirical Bayesian estimation was used to estimate the IIV of individual parameters. The IIV of individual parameters and the correlation of each covariate were analyzed. The following demographic information and laboratory tests were screened and recorded as potential covariates: preoperative disease diagnosis, gender, age, weight, postoperative days (POD), white blood cell (WBC), lymphocyte (LYM), hematocrit (HCT), hemoglobin (HGB), albumin (ALB), total protein (TP), aspartate aminotransferase (AST), alanine transaminase (ALT), total bilirubin (TBIL), direct bilirubin (DBIL), alkaline phosphatase (ALP), urea, creatinine (Cr), and prothrombin time (PT). If the IIV of individual parameters was significantly correlated with the covariates (*p* < 0.05) and was physiologically or clinical pharmacologically significant, then the covariate modeling of these parameter–covariate combinations would be performed using a stepwise approach. The stepwise method included forward inclusion and backward elimination, with a test level α of 0.05 (objective function value (OFV) of 3.84) for forward inclusion and 0.01 (OFV of 6.63) for backward elimination. The final model was developed after excluding all non-significant covariates.

### 2.5 Model evaluation

The goodness-of-fit (GOF) plot and bootstrap method were conducted to evaluate the predictive performances of the final model, wherein GOF was adopted to assess the fitness of the final model to the data, including population predicted concentration-measured value (PRED-DV) scatter plot, individual predicted value-measured value (IPRED-DV) scatter plot, conditional weighted residual–population predicted value (CWRES-PRED) scatter plot, and conditional weighted residual-time (CWRES-TIME) scatter plot. Furthermore, the bootstrap was introduced to test the accuracy and stability of the final model by repeating 1,000 times. The median values and 95% confidence interval (CI, 2.5th–97.5th) of the parameters were calculated and compared with the parameters of the final model.

### 2.6 Simulation of the dosage regimen

The final model was used to perform a simulation to determine the recommended dosage of tacrolimus for both liver cancer and non-liver-cancer populations as per guidance. The C_min_ of tacrolimus at day 10 was simulated after continuous oral administration for 10 days at a dosage of 2.0–6.0 mg BID.

### 2.7 Analysis software

PopPK modeling was performed with a non-linear mixed-effects modeling software (NONMEM, version 7.5.0; ICON Development Solutions, Ellicott City, MD, United States) compiled with GFortran (version 4.6.0, https://gcc.gnu.org/fortran) and interfaced with Perl-speaks-NONMEM (version 5.2.6; https://uupharmacometrics.github.io/PsN). The R software (version 4.2.2; https://www.r-project.org) was adopted to organize the raw data and analyze the NONMEM output.

## 3 Result

### 3.1 Patient characteristics

Data were collected from 196 patients who underwent liver transplantation in Beijing YouAn Hospital of Capital Medical University from November 2021 to December 2023. The study population was divided into liver cancer (N = 118) and non-liver-cancer (N = 78) groups. The demographic and biochemical indicators of these patients are listed in Table 1. Additionally, a total of 802 whole-blood tacrolimus concentrations were recorded. The average concentration was 4.53 ± 3.30 ng/mL, ranging from below the quantification limit (BQL) to 28.8 ng/mL. The histogram of whole-blood tacrolimus concentrations is displayed in Supplementary Figure S1.

**TABLE 1:** Demographic and biochemical characteristics of the patients.

Characteristics	Number or mean (SD)	Median [min, max]
Diagnosis (liver cancer/non-liver-cancer)	118/78	-
Gender (male/female)	148/48	-
Age (year)	54.4 (10.1)	55.0 [16.0, 76.0]
Weight (kg)	70.7 (13.9)	70.0 [41.0, 129]
POD (days)	7.07 (4.92)	5.50 [0.500, 30.9]
WBC (10^9^/L)	7.19 (3.82)	6.43 [1.44, 22.2]
LYM (10^9^/L)	0.718 (0.670)	0.545 [0.0500, 6.45]
HCT (%)	26.8 (5.15)	26.3 [13.5, 40.6]
HGB (g/L)	90.0 (17.2)	87.5 [48.0, 141]
ALB (g/L)	36.2 (4.18)	36.0 [28.4, 57.0]
TP (g/L)	59.6 (8.81)	59.3 [40.4, 139]
AST (U/L)	101 (434)	39.0 [11.0, 5970]
ALT (U/L)	172 (186)	117 [25.0, 1740]
TBIL (μmol/L)	53.8 (62.0)	38.3 [0.290, 457]
DBIL (μmol/L)	36.2 (49.1)	22.1 [3.40, 345]
ALP (U/L)	161 (92.4)	133 [51.0, 570]
Urea (mmol/L)	13.7 (7.73)	11.9 [4.07, 48.1]
Cr (μmol/L)	67.9 (36.1)	60.5 [28.0, 291]
PT (S)	11.8 (2.48)	11.5 [7.90, 27.1]
Daily dose of tacrolimus (mg)	3.90 (0.84)	3.92 [1.40, 6.00]
Concentration of tacrolimus (ng/mL)	4.53 (3.30)	3.80 [BQL, 28.8]

POD, postoperative days; WBC, white blood cell; LYM, lymphocyte; HCT, hematocrit; HGB, hemoglobin; ALB, albumin; TP, total protein; AST, aspartate aminotransferase; ALT, alanine transaminase; TBIL, total bilirubin; DBIL, direct bilirubin; ALP, alkaline phosphatase; Cr, creatinine; PT, prothrombin time.

### 3.2 PopPK modeling

The base model of tacrolimus examined the one-compartment model with linear elimination, the two-compartment model with linear elimination, the one-compartment model plus absorption delay D1, the one-compartment model with nonlinear (Michaelis–Menten) elimination, the one-compartment model with fixed absorption rate constant (Ka) of 0.35 1/h, and the one-compartment model without covariances of η_CL_ and η_VC_. None of the remaining models showed significant improvement compared with the one-compartment model with linear elimination. Thereafter, PK parameters were estimated separately for liver cancer and non-liver-cancer subjects, as listed in Table 2. Although the OFV decreased significantly (−7.50), the estimated parameters were similar in both populations (except the Ka of liver cancer patients was 60.2% higher than that in non-liver-cancer patients), and the IIV of Ka in non-liver-cancer subjects showed great variation, so the separate estimated parameters were not considered.

**TABLE 2:** Estimated PK parameters of patients with or without liver cancer.

Population	CL/F (L^.^h^-1^)	IIV_CL/F	Vc/F (L)	IIV_Vc/F	Ka (1/h)	IIV_Ka
Liver cancer	38.9 (2%)	0.314 (15%)	1603.6 (2%)	0.993 (19%)	0.354 (24%)	3.95 (48%)
Non-liver-cancer	37.7 (3%)	0.528 (24%)	1772.2 (2%)	0.934 (19%)	0.221 (37%)	0.025 (4959%)

CL/F, apparent oral clearance; IIV_CL/F, inter-individual variability of CL/F; Vc/F, apparent volume of distribution; IIV_Vc/F, inter-individual variability of Vc/F; Ka, absorption rate constant (h^-1^); IIV_Ka, inter-individual variability of Ka.

The correlation of 19 selected covariates and IIV was assessed, and nine covariate–PK parameter pairs were stepwise screened to evaluate the influence on the PK behavior of tacrolimus. The final model included weight as the covariate of Vc and DBIL as the covariate of CL. The formula of the final PopPK model of tacrolimus in Chinese liver transplant recipients is as follows.

$${CL}_{i}\left(L/hr\right)=37.6\cdot \exp\left(\eta_{CL,i}\right)\cdot {\left(\frac{DBIL}{22.1}\right)}^{-0.188}.$$

$${VC}_{i}\left(L\right)=1710\cdot \exp\left(\eta_{VC,i}\right)\cdot {\left(\frac{WT}{70}\right)}^{1.4}.$$

$$KA\left(1/hr\right)=0.352\cdot \exp\left(\eta_{KA,i}\right).$$

### 3.3 Model evaluation

**FIGURE 1:**
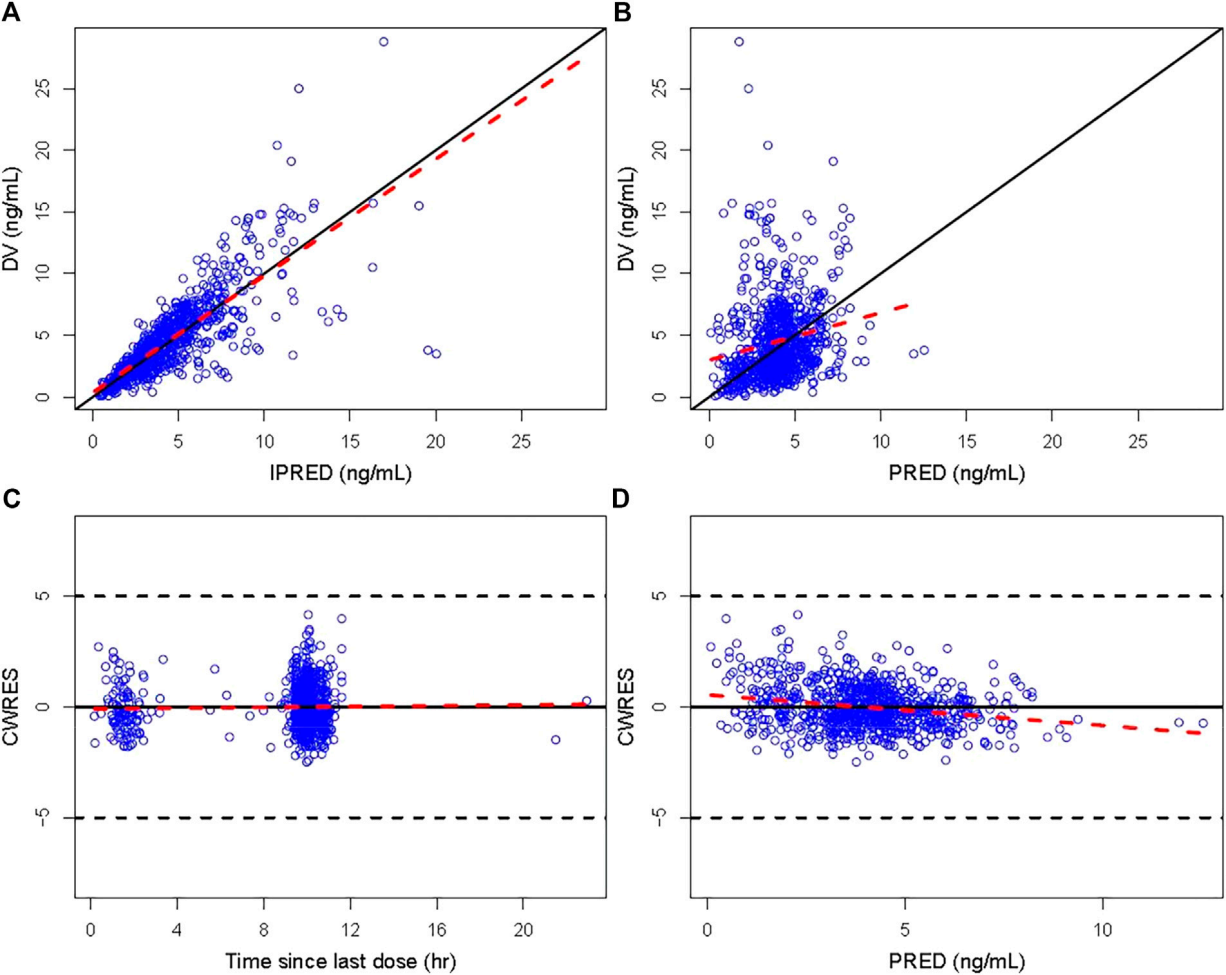
GOF plot of the final model. **(A)** Individual predicted concentration *versus* observed concentration. **(B)** Population predicted concentration *versus* observed concentration. **(C)** CWRES *versus* time since last dose. **(D)** CWRES *versus* population predicted concentration. The solid lines in **(A)** and **(B)** are unity lines, and those in **(C)** and **(D)** represent the zero lines. The red dotted lines are the locally weighted regression Loess lines. CWRES, conditional weighted residuals; IPRED, individual predicted value; PRED, population predicted concentration.

**FIGURE 2:**
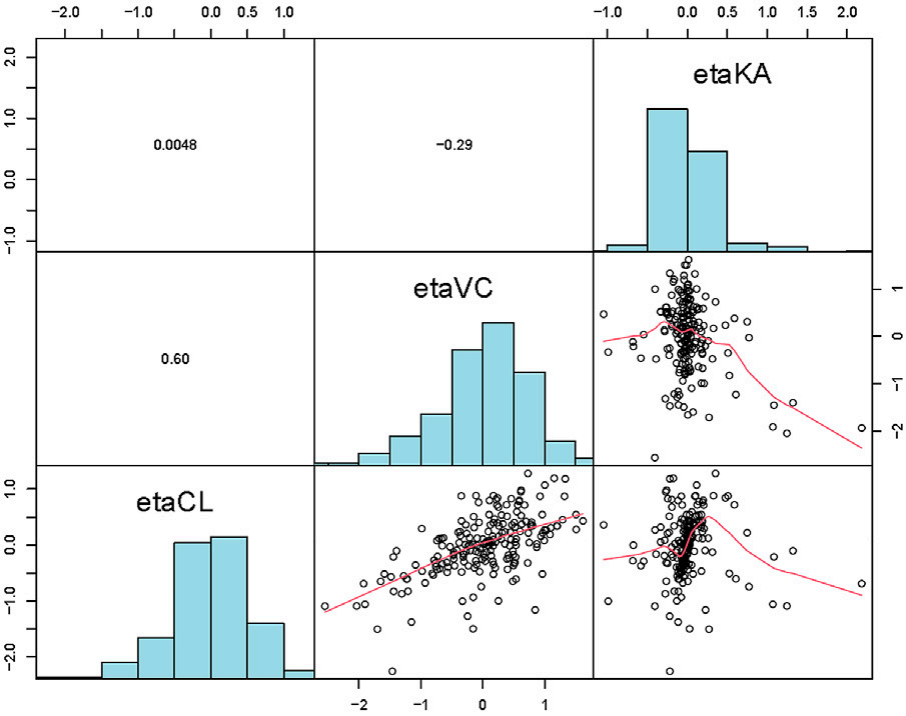
Distribution and correlation of random effects among individuals of the final popPK model.

**FIGURE 3:**
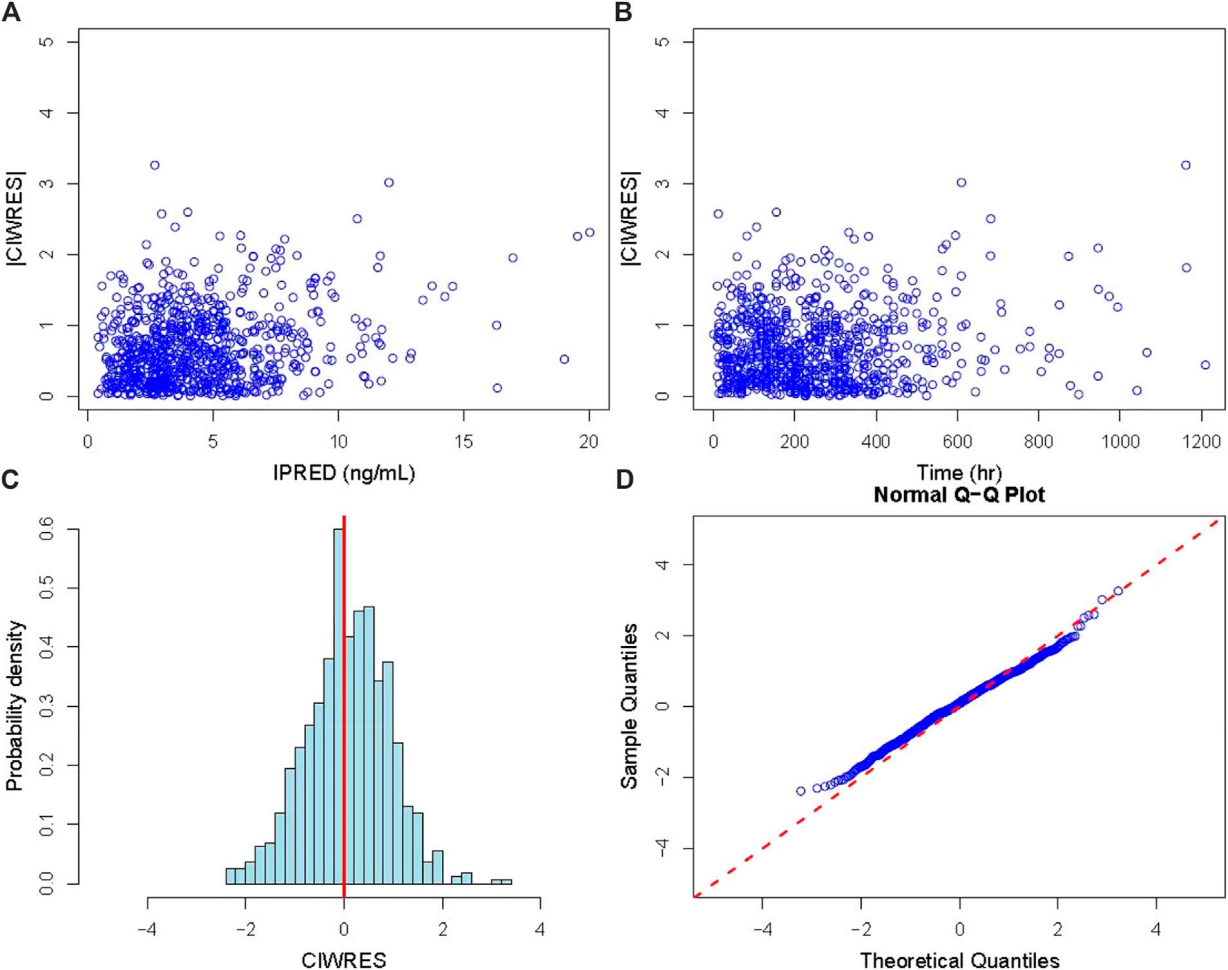
Diagnostic plot of residuals. **(A)** Diagnostic plot of the absolute value of the model’s individual weighted residuals (|CIWRES|) *versus* IPRED; **(B)** diagnostic plot of |CIWRES| *versus* time; **(C)** histogram of the CIWRES where the solid red line is the reference line; **(D)** Q–Q plot of the CIWRES where the dashed red line is the reference line). CIWRES, conditional individual weighted residuals.

The GOF plots of the final PopPK model of tacrolimus are displayed in Figure 1. The data points were symmetrically distributed about the null ordinate, indicating good consistency between the observed and the individual predicted concentrations, although there was some bias between population predicted concentration and observed concentrations. Moreover, η and conditional individual weighted residuals (CIWRES) were all uniformly distributed around 0. All the results suggested the good predictive performance of the final model with an acceptable bias (Figures 2, 3).

The final model was further validated based on a bootstrap approach. The deviations between the calculated median values of PK parameters in the bootstrap datasets and the estimated values of the final PopPK model were all below 4%. The estimated values of these parameters were all within the 95% prediction intervals (PI) derived from the bootstrap (Table 3), and the bootstrap success rate was 90.5%. The outcome demonstrated that the obtained PopPK model possesses excellent accuracy and stability, which could lead to an acceptable predictive capability of the PK profile. Furthermore, the graph of prediction-correct visual predictive check also indicated that the final model can effectively describe the central trends and variations in pharmacokinetic data (Supplementary Figure S2).

**TABLE 3:** PopPK parameters estimated from the final model and bootstrap validation.

Parameters	Final model		Bootstrap	
	Estimate	95% CI	Median	95% PI
CL/F (L/h)	37.6	34–41.7	37.7	33.9–41.8
Vc/F (L)	1710	1400–2100	1690	1380–2060
Ka (1/h)	0.352	0.15–0.826	0.339	0.166–1.26
Effect of WT on CL/F	−0.188	−0.315 ∼ −0.0616	−0.192	−0.321 ∼ −0.0553
Effect of DBIL on Vc/F	1.4	0.512–2.28	1.41	0.403–2.29
IIV_CL/F (%)	66.6	55–77.3	66	54.5–76.1
IIV_Vc/F (%)	126	95–158	122	95.6–154
IIV_Ka (%)	236	Na ∼1070	236	44.3–1700
Covariance of IIV_CL/F and IIV_Vc/F	0.226	0.105–0.348	0.222	0.0997–0.353
Residual variability				
Proportional (%)	35.6	32–38.9	35.4	31.9–38.8
Additive (μg/L)	0.355	0.166–0.473	0.359	0.209–0.487

### 3.4 Simulations

**FIGURE 4:**
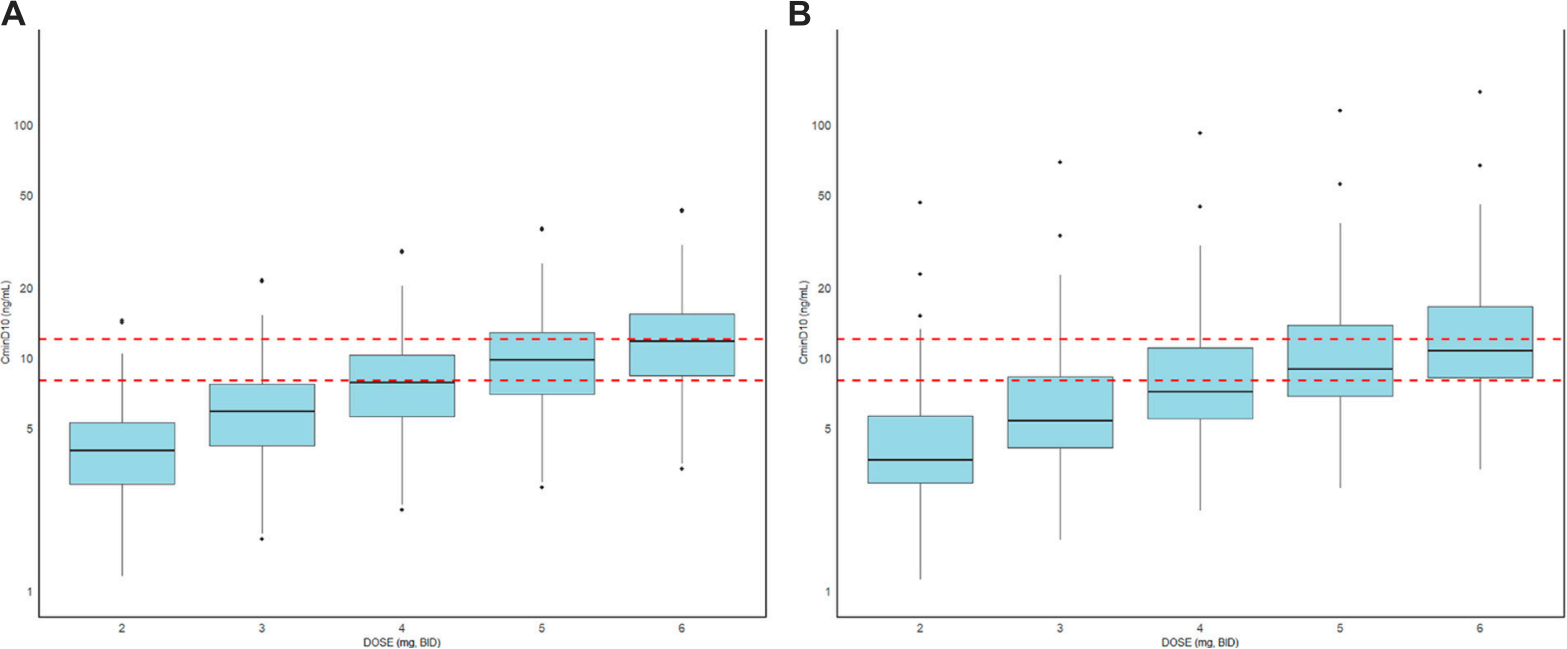
Box plots of the distributions of simulated C_min_ on the 10th day of tacrolimus administration in patients with **(A)** or without liver cancer **(B)** before surgery at a dose of 2.0–6.0 mg BID. The horizontal bars in the middle reflect the median C_min_, and the outer boundaries of the boxes indicate the ranges of the 25th and 75th percentiles. The red dotted lines represent 8.0–12.0 ng/mL as the trough concentration recommended by tacrolimus guidance.

The exposure of tacrolimus in patients with or without liver cancer before surgery was simulated after continuous oral administration for 10 days at a dose of 2.0–6.0 mg BID, and the result is shown in Table 4 and Figure 4. The median (25th–75th quantiles, P25–P75) of C_min_ at the 10th day in the liver cancer group was higher than that of the non-liver-cancer group at all the administrated dosages. However, the deviations of the estimated C_min_ at the 10th day between the two populations were all below 10%, demonstrating the relatively minor difference in the PK behavior of tacrolimus in liver cancer and non-liver-cancer populations based on the available data in this study. To achieve a trough concentration of 8.0–12.0 ng/mL at an early stage after liver transplant, as recommended by tacrolimus guidance, 5.0 mg BID dosage was required for both groups.

**TABLE 4:** Simulation of C_min_ at day 10 under the dosage of 2.0–6.0 mg BID of tacrolimus in liver cancer and non-liver-cancer groups.

Dose (mg)	Median (P_25_–P_75_)		Deviation
	Liver cancer	Non-liver-cancer	
2.0	3.99 (2.86–5.26)	3.66 (2.91–5.61)	0.33 (8.3%)
3.0	5.88 (4.18–7.70)	5.36 (4.11–8.28)	0.52 (8.8%)
4.0	7.84 (5.57–10.3)	7.15 (5.48–11.0)	0.69 (8.8%)
5.0	9.80 (6.96–12.8)	8.93 (6.84–13.8)	0.87 (8.9%)
6.0	11.8 (8.35–15.4)	10.7 (8.21–16.6)	1.10 (9.3%)

## 4 Discussion

After liver transplantation, tacrolimus is usually required to prevent rejection and enhance the survival. However, the narrow treatment range and high variability of tacrolimus largely obstruct its wide application in clinical practice (Kirubakaran et al., 2020). To date, despite numerous PopPK models having characterized the impact of demographic and biochemical indicators, such as weight, hemoglobin, alanine aminotransferase, and bilirubin, on the PK behavior of tacrolimus, few have focused on whether the indication for transplantation is a possible explanation for PK variability due to the varied compositions of patients in different studies (Cai et al., 2022; Li et al., 2023; Ponthier et al., 2023).

Liver cancer is a primary contributor to tumor and tumor-related death in China and worldwide. Liver transplantation is offered as a cure for liver cancer (Yankol et al., 2024). However, liver cancer recurrence after liver transplantation is still a critical clinical challenge, with reported rates of approximately 10%–20% in the first year after liver transplantation (To et al., 2024). The recurrence mainly occurs due to the potential effects of the accumulated genetic mutations on the liver cells. Whether the sustained effect will have an impact on the metabolism of tacrolimus *in vivo* has not been fully elucidated. Therefore, the current study included 807 observable concentrations from 196 subjects for PopPK analysis to investigate the PK differences of tacrolimus between liver cancer and non-liver-cancer populations. A one-compartment pharmacokinetic model with first-order absorption and elimination was finally chosen to describe the *in vivo* process of tacrolimus, incorporating DBIL as a significant covariate of CL and weight as a significant covariate of Vc.

The parameters of the established PopPK model were comparable to the previously reported parameters. Although Ka, which was estimated to be 0.352 h^−1^, was lower than the common Ka (4.48 h^−1^) for tacrolimus (Kirubakaran, et al., 2020), it was similar to the Ka reported in a Belgian renal transplantation research (0.45 h^−1^) (Musuamba, et al., 2012) and two PopPK models for Chinese liver transplant recipients (0.55 h^−1^ and 0.419 h^−1^) (Lu et al., 2015; Chen et al., 2017). For CL/F and Vc/F, which had a large degree of variation in different studies, the estimations in this study were also in the same order of magnitude as those of the one-compartment models developed in other published research studies (Fukatsu et al., 2001; Zhao et al., 2013).

The effects of DBIL on CL/F and weight on Vc/F found in this study were two common covariates of PopPK models for tacrolimus (Kirubakaran, et al., 2020). Nevertheless, some research studies have reported that POD may be a possible explanation for PK variability of tacrolimus. However, based on our study result, the effect of POD on PK was not observed, which may be because the collected clinical data were from the early postoperative period, and longer follow-up data are needed to determine the impact of POD on the PK characteristics of tacrolimus.

This study found that CL and Vc both showed no significant differences between the liver cancer and non-liver-cancer groups. Although the Ka of patients with liver cancer was 60.2% higher than that in non-liver-cancer patients, the IIV of Ka was incalculable. This could be attributed to the fact that the clinical data collected in this study were of the early stage of liver transplantation, when the PK differences between liver cancer and non-liver-cancer populations had not yet fully emerged, and most of the concentrations were trough concentrations, resulting in a more difficult estimation of Ka.

Since the PK parameters of tacrolimus had little difference in the liver cancer patients compared with those with other liver diseases, the exposure of tacrolimus in these two groups was further simulated. Our study found that after oral administration of different doses of tacrolimus, the exposure had increased a little in the liver cancer groups. Although the increase was less than 10%, more attention should be paid to the tumor-induced liver transplantation for a more precise therapy.

However, there are still some limitations in our study. First, the CYP3A5 gene polymorphism, which is a potential covariate, was not included in our model (Zhu, et al., 2015; Dong, et al., 2022). The influence of this factor on the PK behavior of tacrolimus needs to be determined in the future. Second, more follow-up data will be incorporated to improve the accuracy of the final model in predicting the concentration. Third, more subgroups will be included to provide more references for the clinical application of tacrolimus.

## 5 Conclusion

In conclusion, we developed a reliable PopPK model for Chinese adult liver transplant recipients who received tacrolimus. In this PopPK model, we revealed a significant effect of weight and DBIL on the PK of tacrolimus. Moreover, the PK parameters of tacrolimus in both liver cancer and non-liver-cancer populations were compared. There was no significant difference in CL and Vc between the two groups, and Ka was found to be 60% higher in liver cancer populations. Due to the limitation of the current study, more data are warranted to further investigate the differences of the PK of tacrolimus in these two populations.

## Data Availability

The original contributions presented in the study are included in the article/Supplementary Material; further inquiries can be directed to the corresponding authors.
