# Supplementary material for: Population pharmacokinetics study of tacrolimus in liver transplant recipients: a comparison between patients with or without liver cancer before surgery
Source: Front Pharmacol. 2024 Aug 23;15:1449535. doi: 10.3389/fphar.2024.1449535 (PMC11385303; doi:10.3389/fphar.2024.1449535)
Supplement: Supplementary file 1 [file DataSheet2.doc]

**Population pharmacokinetics study of tacrolimus in liver transplant recipients: a comparison between patients with or without liver cancer before surgery**

Haihong Bai ^1^, Juping Yun ^1^, Zihe Wang ^1^, Yingmin Ma ^2*^, Wei Liu ^1*^

^1^ Department of Pharmacy, Beijing YouAn Hospital of Capital Medical University,

Beijing 100069, PR China

^2^ Department of Respiratory and Critical Care Medicine, Beijing YouAn Hospital of Capital Medical University, Beijing 100069, PR China.

* Corresponding author: Yingmin Ma ^*^, E-mail: [ma.yingmin@163.com;](mailto:ma.yingmin@163.com;) Wei Liu ^*^, E-mail: [liuwei8090@126.com.](mailto:liuwei8090@126.com;)


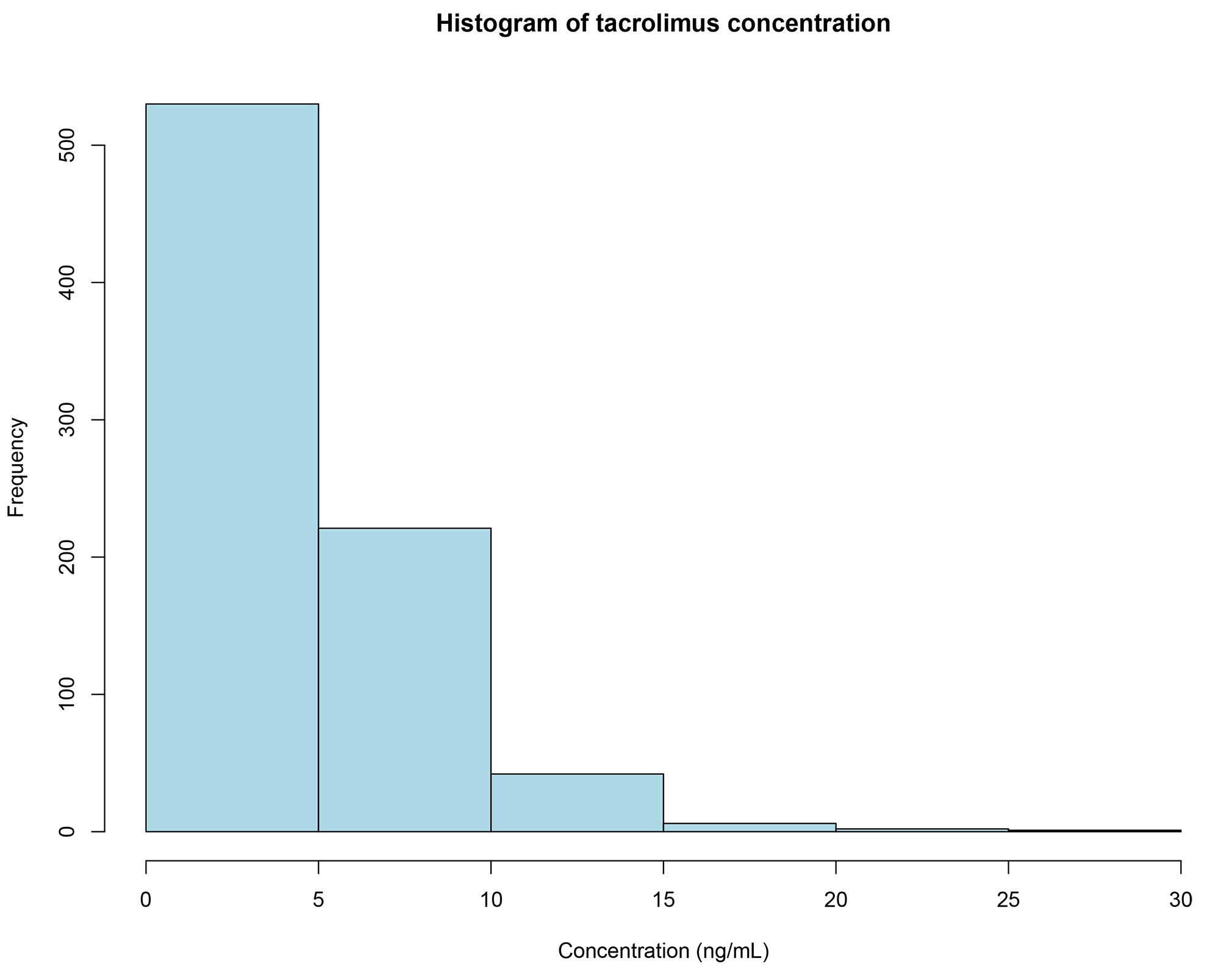
Fig. S1 The histogram of whole blood tacrolimus concentrations


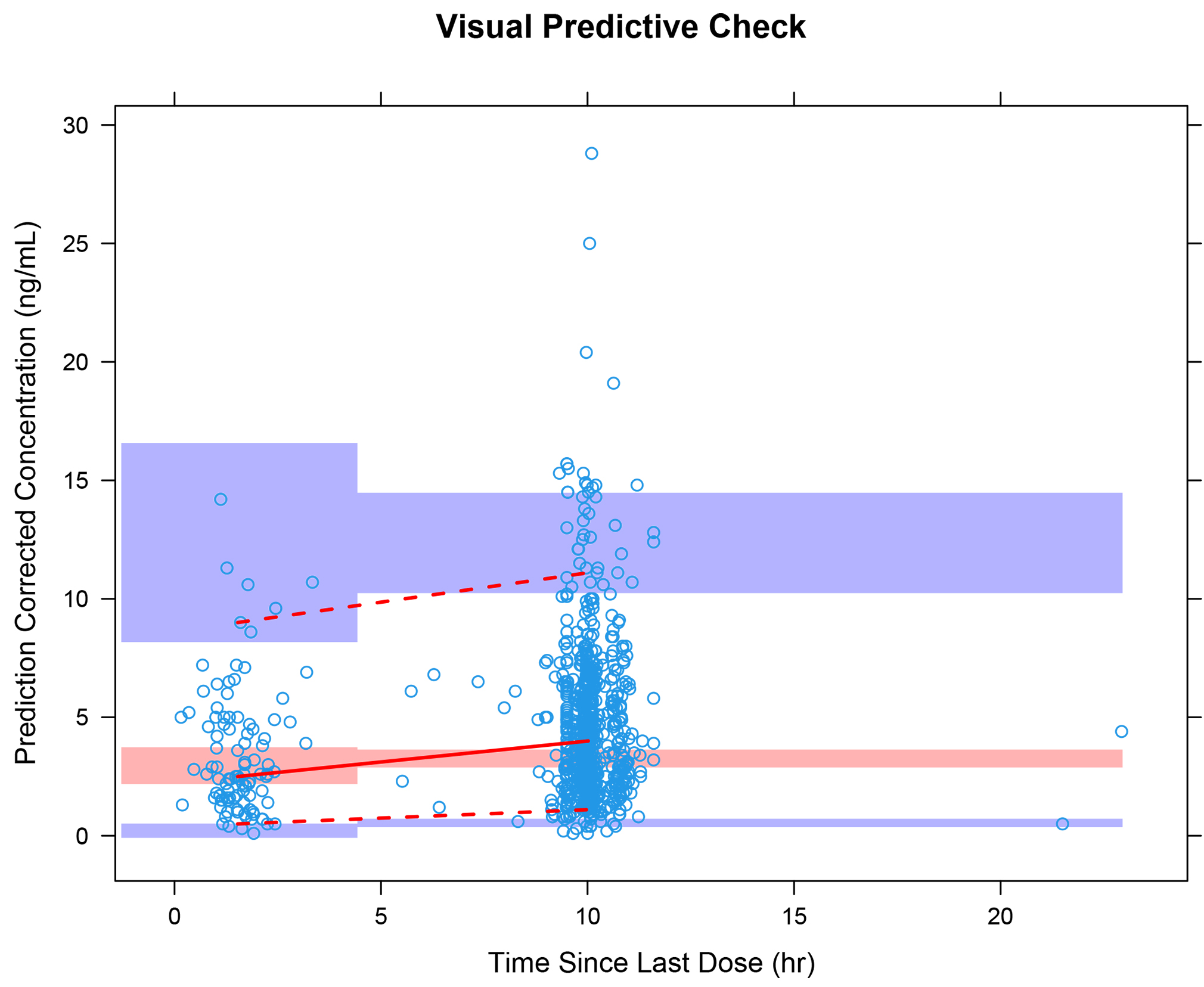
Fig. S2 The graph of Prediction-Correct Visual Predictive Check. The red shading in the graph shows the 95% confidence interval for the median of the predicted values, and the solid red line shows the median of the measured values. The blue shading shows the 95% confidence intervals for the 2.5% and 97.5% quartiles of the predicted values, respectively, and the red dashed line shows the 2.5% and 97.5% quartiles of the measured values for each interval.
