# Supplementary material for: Population pharmacokinetics study of tacrolimus in liver transplant recipients: a comparison between patients with or without liver cancer before surgery
Source: Front Pharmacol. 2024 Aug 23;15:1449535. doi: 10.3389/fphar.2024.1449535 (PMC11385303; doi:10.3389/fphar.2024.1449535)
Supplement: Supplementary file 2 [file DataSheet1.docx]

**NONMEM code of Base model 01:**

;; 1. Based on:

;; 2. Description: 1CMT with linear elimination

$SIZE PD=100

$PROBLEM PopPK of TKMS

$INPUT C ROW ID SUBJID=DROP GROUP DOSE DOSEFRQ NTIME=DROP TIME TSLD RATE1 RATE2 AMT DV LDV CMT BLQ MDV EVID LLOQ

SEX AGE HT WT Diagnosis=DROP AMLZ PNSL MKFN KBFJ LTTIME SGTIME Diagnosis1 WBC GRA LYMN MONO EOS BASO RBC

Hb HCT MCV MCH MCHC PLT ALT AST TBIL DBIL TP ALB CLB GGT ALP TBA BUN CREAT GFR UA CO2CP PT PR PTR INR APTT

APTTR FIB TT DD FDP AT TLR CD3 CD8LR CD8 CD4LR CD4 LYM CD4CD8R BMI BSA CRCL

$DATA dataset-TKMS.csv IGNORE=C IGNORE(C.GT.0)

$SUBROUTINES ADVAN2 TRANS2

$PK

CL = EXP(THETA(1) + ETA(1))

V = EXP(THETA(2) + ETA(2))

KA = EXP(THETA(3) + ETA(3))

S2 = V/1000

$ERROR

;IPRED = LOG(F+0.00001)

;Y = IPRED + EPS(1)

IPRED = F

Y = IPRED * (1 + EPS(1)) + EPS(2)

$THETA

(-10,3.6,10) ;1 CL(L/hr)

(-10,7.5,10) ;2 VC(L)

(-10,-1.2,10) ;3 KA(1/hr)

$OMEGA BLOCK(2)

0.39 ;1 IIV_CL

0.21 1.06 ;2 IIV_VC

$OMEGA

1.65 ;3 IIV_KA

$SIGMA

0.127 ;1 PROP

0.115 ;2 ADD

$ESTIMATION METHOD=1 INTER POSTHOC MAXEVAL=9999 NOABORT PRINT=5 NSIG=3 SIGL=9

$COVARIANCE PRINT=E

$TABLE ROW DOSE ID TIME TSLD DV AMT CMT BLQ LLOQ MDV EVID

SEX AGE HT WT AMLZ PNSL MKFN KBFJ LTTIME SGTIME Diagnosis1 WBC GRA LYMN MONO EOS BASO RBC

Hb HCT MCV MCH MCHC PLT ALT AST TBIL DBIL TP ALB CLB GGT ALP TBA BUN CREAT GFR UA CO2CP PT PR PTR INR APTT

APTTR FIB TT DD FDP AT TLR CD3 CD8LR CD8 CD4LR CD4 LYM CD4CD8R BMI BSA CRCL

CWRES CIWRES PRED IPRED CL V KA ETA1 ETA2 ETA3

NOPRINT ONEHEADER FILE=model01.RES

**NONMEM code of Final model:**

;; 1. Based on: model24

;; 2. Description: final model

;; x1. Author: C04468

$SIZE PD=100

$PROBLEM PopPK of TKMS

$INPUT C ROW ID SUBJID=DROP GROUP DOSE DOSEFRQ NTIME=DROP TIME TSLD RATE1 RATE2 AMT DV LDV CMT BLQ MDV EVID LLOQ

SEX AGE HT WT Diagnosis=DROP AMLZ PNSL MKFN KBFJ LTTIME SGTIME Diagnosis1 WBC GRA LYMN MONO EOS BASO RBC

Hb HCT MCV MCH MCHC PLT ALT AST TBIL DBIL TP ALB CLB GGT ALP TBA BUN CREAT GFR UA CO2CP PT PR PTR INR APTT

APTTR FIB TT DD FDP AT TLR CD3 CD8LR CD8 CD4LR CD4 LYM CD4CD8R BMI BSA CRCL

$DATA dataset-TKMS.csv IGNORE=C IGNORE(C.GT.0)

$SUBROUTINES ADVAN2 TRANS2

$PK

WTI = WT

BSAI = BSA

WBCI = WBC

GRAI = GRA

MCVI = MCV

MCHCI = MCHC

TBILI = TBIL

DBILI = DBIL

IF (WTI.LT.0) WTI = 70

IF (BSAI.LT.0) BSAI = 1.8

IF (WBCI.LT.0) WBCI = 6.425

IF (GRAI.LT.0) GRAI = 5.08

IF (MCVI.LT.0) MCVI = 94.8

IF (MCHCI.LT.0) MCHCI = 335

IF (TBILI.LT.0) TBILI = 38.3

IF (DBILI.LT.0) DBILI = 22.1

CLDBIL = THETA(4)*LOG(DBILI/22.1)

CLCOV = CLDBIL

V1WT = THETA(5)*LOG(WTI/70)

V1COV = V1WT

CL = EXP(THETA(1) + ETA(1) + CLCOV)

V = EXP(THETA(2) + ETA(2) +V1COV)

KA = EXP(THETA(3) + ETA(3))

S2 = V/1000

$ERROR

;IPRED = LOG(F+0.00001)

;Y = IPRED + EPS(1)

IPRED = F

Y = IPRED * (1 + EPS(1)) + EPS(2)

$THETA

(-10,3.6,10) ;1 CL(L/hr)

(-10,7.5,10) ;2 VC(L)

(-10,-1.2,10) ;3 KA(1/hr)

(-10,0.1,10) ;4 CLDBIL

(-10,0.1,10) ;5 V1WT

$OMEGA BLOCK(2)

0.39 ;1 IIV_CL

0.21 1.06 ;2 IIV_VC

$OMEGA

1.65 ;3 IIV_KA

$SIGMA

0.127 ;1 PROP

0.115 ;2 ADD

$ESTIMATION METHOD=1 INTER POSTHOC MAXEVAL=9999 NOABORT PRINT=5 NSIG=3 SIGL=9

$COVARIANCE PRINT=E

$TABLE ROW DOSE ID TIME TSLD DV AMT CMT BLQ LLOQ MDV EVID

SEX AGE HT WT AMLZ PNSL MKFN KBFJ LTTIME SGTIME Diagnosis1 WBC GRA LYMN MONO EOS BASO RBC

Hb HCT MCV MCH MCHC PLT ALT AST TBIL DBIL TP ALB CLB GGT ALP TBA BUN CREAT GFR UA CO2CP PT PR PTR INR APTT

APTTR FIB TT DD FDP AT TLR CD3 CD8LR CD8 CD4LR CD4 LYM CD4CD8R BMI BSA CRCL

CWRES CIWRES PRED IPRED CL V KA ETA1 ETA2 ETA3

NOPRINT ONEHEADER FILE=modelFL.RES

**R code of review of PopPK model:**

# Environment settings ####

rm(list=ls())

DIRscript <- dirname(rstudioapi::getActiveDocumentContext()$path)

setwd(dir = DIRscript)

DIRproject <- "../"

DIRout <- "../result/pk/"

DIRmodel <- "../pk/"

library(tidyverse)

# functions ####

f.INDI <- function(no.model){

RES <- read.csv(paste(DIRmodel,"model",no.model,".RES",sep = ""),skip = 1,sep = "")

data <- RES[RES$MDV==0,]

pdf(paste(DIRout,"Figure . Individual fit plot",no.model,".pdf",sep = ""),height = 15,width = 9)

par(mfrow=c(4,3),mar=c(2,2,2,1),oma=c(4,4,5,1))

for (i in 1:length(unique(data$ID))){

if (i<=208){

datai <- data[data$ID==unique(data$ID)[i],]

plot(datai$DV~datai$TIME,log="",xlab="n",ylab="n",col="blue",

ylim=c(min(datai$DV,datai$PRED,datai$IPRED)*0.8,

max(datai$DV,datai$PRED,datai$IPRED)*1.25))

lines(datai$PRED~datai$TIME,col="black",lty=2)

lines(datai$IPRED~datai$TIME,col="red",lty=1)

mtext(unique(data$ID)[i],side = 3,line = 0)

}else{

datai <- data[data$ID==unique(data$ID)[i],]

plot(datai$DV~datai$TIME,log="",xlab="n",ylab="n",col="blue",

ylim=c(0,

max(datai$DV,datai$PRED,datai$IPRED)*1.25))

lines(datai$PRED~datai$TIME,col="black",lty=2)

lines(datai$IPRED~datai$TIME,col="red",lty=1)

mtext(unique(data$ID)[i],side = 3,line = 0)

}

if (i %% 12==1){

mtext("Time (hr)",line = 1.5,cex=1.25,outer = T,side = 1)

mtext("Concentration (ng/mL)",line = 1.5,cex=1.25,outer = T,side = 2)

mtext("Circle: DV; Dashed black line: PRED; Solid red line: IPRED",line = .5,cex=1.25,outer = T,side = 3)

}

}

dev.off()

}

f.GOF <- function(no.model){

RES <- read.csv(paste(DIRmodel,"model",no.model,".RES",sep = ""),skip = 1,sep = "")

data <- RES[RES$MDV==0,]

pdf(paste(DIRout,"Figure . GOF plot",no.model,".pdf",sep = ""),height = 8,width = 10)

par(mfrow=c(2,2),mar=c(3,3,2,1),oma=c(1,1,1,1))

lim1 <- range(data$DV,data$IPRED,data$PRED)

plot(y=data$DV,x=data$IPRED,ylab="",xlab="",xaxt="n",yaxt="n",col="blue",log="",xlim=lim1,ylim=lim1)

lim1

axis(side = 1,at = seq(0,30,5),labels = seq(0,30,5))

axis(side = 2,at = seq(0,30,5),labels = seq(0,30,5))

abline(a=0,b=1,col="black",lwd=2)

y=data$DV;x=data$IPRED

FIT <- lm(y ~ x)

# xp=seq(range(x)[1],range(x)[2],0.01)

xp=range(data$DV,data$IPRED)

pred <- predict(FIT,newdata=data.frame(x=xp))

lines(pred~xp,lwd=3,lty=2,col="red")

mtext(side=1,"IPRED (ng/mL)",line=2.5)

mtext(side=2,"DV (ng/mL)",line=2.5)

plot(y=data$DV,x=data$PRED,ylab="",xlab="",xaxt="n",yaxt="n",col="blue",log="",xlim=lim1,ylim=lim1)

lim1

axis(side = 1,at = seq(0,30,5),labels = seq(0,30,5))

axis(side = 2,at = seq(0,30,5),labels = seq(0,30,5))

abline(a=0,b=1,col="black",lwd=2)

y=data$DV;x=data$PRED

FIT <- lm(y ~ x)

#xp=seq(range(x)[1],range(x)[2],0.01)

xp=range(x)

pred <- predict(FIT,newdata=data.frame(x=xp))

lines(pred~xp,lwd=3,lty=2,col="red")

mtext(side=1,"PRED (ng/mL)",line=2.5)

mtext(side=2,"DV (ng/mL)",line=2.5)

plot(y=data$CWRES,x=data$TSLD,ylab="",xlab="",xaxt="n",yaxt="n",ylim=c(-8,8),col="blue")

axis(side = 1,at = seq(0,24,4),labels = seq(0,24,4))

axis(side = 2,at = seq(-5,5,5))

abline(h=c(-5,0,5),col="black",lty=c(2,1,2),lwd=2)

y=data$CWRES;x=data$TSLD

FIT <- lm(y ~ x)

xp=range(x)

pred <- predict(FIT,newdata=data.frame(x=xp))

lines(pred~xp,lwd=3,lty=2,col="red")

mtext(side=1,"Time since last dose (hr)",line=2.5)

mtext(side=2,"CWRES",line=2.5)

plot(y=data$CWRES,x=data$PRED,ylab="",xlab="",xaxt="n",yaxt="n",ylim=c(-8,8),col="blue")

axis(side = 1,at = seq(0,30,5),labels = seq(0,30,5))

axis(side = 2,at = seq(-5,5,5))

abline(h=c(-5,0,5),col="black",lty=c(2,1,2),lwd=2)

y=data$CWRES;x=data$PRED

FIT <- lm(y ~ x)

xp=range(x)

pred <- predict(FIT,newdata=data.frame(x=xp))

lines(pred~xp,lwd=3,lty=2,col="red")

mtext(side=1,"PRED (ng/mL)",line=2.5)

mtext(side=2,"CWRES",line=2.5)

options(scipen=999)

dev.off()

}

# GOF ####

f.GOF("01")

f.GOF("02")

#f.GOF("03")

#f.GOF("04")

f.GOF("05")

f.GOF("06")

f.GOF("24")

# Individual fit plot ####

#not applicable

f.INDI("01")

f.INDI("02")

#f.INDI("03")

#f.INDI("04")

f.INDI("05")

f.INDI("06")

f.INDI("24")

# COV base ####

no.model <- "01"

RES <- read.csv(paste(DIRmodel,"model",no.model,".RES",sep = ""),skip = 1,sep = "")

data <- RES[RES$MDV==0,]

base <- data[!duplicated(data$ID),]

eta <- c("ETA1","ETA2","ETA3")

eta.NAME <- c("etaCL","etaV","etaKA")

head(base)

catcov=c("SEX", "AMLZ","PNSL","MKFN","KBFJ","Diagnosis1")

contcov=c("AGE","HT","WT","BMI","BSA","CRCL","LTTIME","SGTIME",

"WBC","GRA","LYMN","MONO","EOS", "BASO", "RBC", "Hb", "HCT",

"MCV", "MCH", "MCHC", "PLT", "ALT", "AST", "TBIL", "DBIL", "TP",

"ALB", "CLB", "GGT", "ALP", "TBA", "BUN", "CREAT", "GFR", "UA", "CO2CP",

"PT", "PR", "PTR", "INR", "APTT", "APTTR", "FIB", "TT", "DD", "FDP",

"AT", "TLR", "CD3", "CD8LR", "CD8", "CD4LR", "CD4", "LYM", "CD4CD8R")

netas <- length(eta)

pdf(paste(DIRout,"Figure . Covariate Evaluations Based on the Base Model",no.model,".pdf",sep=""),width=9,height=6)

PvalResultsCat=matrix(0,length(catcov),netas+1)

par(mfrow=c(2,3),mar=c(4,5,2,1),oma=c(2,2,4,1))

for (i in 1:length(catcov)){

xxi=base[,c(catcov[i],eta)]

names(xxi)[1]="cov"

xxi=xxi[!is.na(xxi$cov),]

xxi=xxi[xxi$cov!="NA",]

xxi=xxi[xxi$cov!="-99",]

xxi$cov=as.character(xxi$cov)

names=sort(unique(xxi$cov))

x=xxi$cov

unique(xxi$cov)

pvali <- c()

for (j in 1:length(eta.NAME)){

y=xxi[,eta[j]]

boxplot(split(y,x),ylab=eta.NAME[j],xlab=catcov[i],names=names,cex=1.25,ylim=c(min(y)*1.1,max(y)*1.3),col="blue",medcol="white",cex.axis=1.5,cex.lab=2)

lm.fit1=lm(y~as.factor(x))

lm.fit2=lm(y~1)

pval=round(anova(lm.fit2,lm.fit1)[6][2,],4)

n1 <- tapply(x,x,length)

text(rep(max(y)*1.3,length(unique(x))),paste("n=",n1 ) )

title(paste("p value=",pval," (N=",nrow(xxi),")",sep=""),cex.main=1.5)

pvali=c(pvali,pval)

}

PvalResultsCat[i,]=c(pvali,nrow(xxi))

}

colnames(base)

PvalResultsCont=matrix(0,length(contcov),netas+1)

par(mfrow=c(2,3),mar=c(4,5,2,1),oma=c(2,2,4,1))

for (i in 1:length(contcov)){

xxi=base[,c(contcov[i],eta)]

names(xxi)[1]="cov"

xxi$cov=as.numeric(as.character(xxi$cov))

xxi=xxi[xxi$cov>=0,]

xxi$cov=as.double(as.character(xxi$cov))

xxi=xxi[!is.na(xxi$cov),]

xsim=c(min(xxi$cov,na.rm=T),max(xxi$cov,na.rm=T))

pvali <- c()

for (j in 1:length(eta.NAME)){

plot(xxi$cov,xxi[,eta[j]],ylab=eta.NAME[j],xlab=contcov[i],ylim=c(-1.5,1.5),cex=1.15,cex.axis=1.5,cex.lab=2)

lm.fit=lm(xxi[,eta[j]]~xxi$cov,na.action=na.exclude)

xsim <- c(min(xxi$cov),max(xxi$cov))

lines(xsim,coef(lm.fit)[1]+xsim*coef(lm.fit)[2],lwd=4,col="blue")

pval=round(anova(lm.fit)[5][1,],4)

title(paste("p value=",pval," (N=",nrow(xxi),")",sep=""),cex.main=1.5)

pvali=c(pvali,pval)

}

#对单个协变量的相关性分析

PvalResultsCont[i,]=c(pvali,nrow(xxi))

}

dev.off()

PvalResults <- rbind(PvalResultsCont,PvalResultsCat)

PvalResults <- data.frame(PvalResults)

colnames(PvalResults) <- c(eta.NAME,"N")

row.names(PvalResults) <- c(contcov,catcov)

PvalResults$ROW <- 1:nrow(PvalResults)

write.csv(PvalResults,paste(DIRout,"Table . COV analysis ",no.model,".csv",sep = ""))

base$AGE[base$AGE>0] %>% median

base$WT[base$WT>0] %>% median

base$BMI[base$BMI>0] %>% median

base$BSA[base$BSA>0] %>% median

base$CRCL[base$CRCL>0] %>% median

base$TBIL[base$TBIL>0] %>% median

base$DBIL[base$DBIL>0] %>% median

base$WBC[base$WBC>0] %>% median

base$GRA[base$GRA>0] %>% median

base$MCV[base$MCV>0] %>% median

base$MCHC[base$MCHC>0] %>% median

sum(base$WT==-99)/length(base$ROW)

sum(base$BSA==-99)/length(base$ROW)

sum(base$WBC==-99)/length(base$ROW)

sum(base$GRA==-99)/length(base$ROW)

sum(base$MCV==-99)/length(base$ROW)

sum(base$MCHC==-99)/length(base$ROW)

sum(base$TBIL==-99)/length(base$ROW)

sum(base$DBIL==-99)/length(base$ROW)

# CIWRES plots ####

no.model <- "FL"

RES <- read.csv(paste(DIRmodel,"model",no.model,".RES",sep = ""),skip = 1,sep = "")

data <- RES[RES$MDV==0,]

pdf(paste(DIRout,"Figure . Plots of CIWRES",no.model,".pdf",sep = ""),height = 8,width = 10)

par(mfrow=c(2,2),mar=c(3,3,2,1),oma=c(1,1,1,1))

plot(y=abs(data$CIWRES),x=data$IPRED,ylab="",xlab="",xaxt="n",yaxt="n",col="blue",log="",ylim=c(0,5))

axis(side = 1,at = seq(0,30,5),labels = seq(0,30,5))

axis(side = 2,at = seq(0,5,1),labels = seq(0,5,1))

mtext(side=1,"IPRED (ng/mL)",line=2.5)

mtext(side=2,"|CIWRES|",line=2.5)

plot(y=abs(data$CIWRES),x=data$TIME,ylab="",xlab="",yaxt="n",ylim=c(0,5),col="blue")

axis(side = 2,at = seq(0,5,1))

mtext(side=1,"Time (hr)",line=2.5)

mtext(side=2,"|CIWRES|",line=2.5)

hist(data$CIWRES,main = "",col = "lightblue",xlim = c(-5,5),freq = F,breaks = 25)

abline(v=0,col="red",lty=1,lwd=2)

mtext(side=1,"CIWRES",line=2.5)

mtext(side=2,"Probability density",line=2.5)

qqnorm(y = data$CIWRES,col="blue",ylab="",xlab="",plot.it = T,datax = F,xlim=c(-5,5),ylim=c(-5,5))

abline(a=0,b=1,lty=2,lwd=2,col="red")

mtext(side=1,"Theoretical Quantiles",line=2.5)

mtext(side=2,"Sample Quantiles",line=2.5)

dev.off()

# base model parameter ####

no.model <- "01"

ext <- read.csv(paste(DIRmodel,"model",no.model,".ext",sep = ""),skip = 1,sep = "")

parameter <- t(ext[ext$ITERATION=="-1000000000",])

se <- t(ext[ext$ITERATION=="-1000000001",])

out <- data.frame(parameter=parameter[,1],se=se[,1])

out <- out[2:(nrow(out)-1),]

out <- out[out$parameter!=0,]

out <- out[out$parameter!=1,]

out$rse <- out$se/out$parameter*100

out$row <- 1:nrow(out)

out$parameter[c(1:3)] <- exp(out$parameter[c(1:3)])

out$parameter[c(6,8,9)] <- sqrt(exp(out$parameter[c(6,8,9)])-1)*100

out$parameter[c(4)] <- sqrt(out$parameter[c(4)])*100

out$parameter[c(5)] <- sqrt(out$parameter[c(5)])

out$rse[c(1:3)] <- sqrt(exp(out$se[c(1:3)]^2)-1)*100

out$rse[c(4:6,8,9)] <- out$rse[c(4:6,8,9)]/2

out$par.rse <- paste(signif(out$parameter,3)," (",signif(out$rse,3),")",sep = "")

shr <- read.csv(paste(DIRmodel,"model",no.model,".lst",sep = ""))

eps.shr <- shr[grepl(shr[,1],pattern = "EPSSHRINKSD"),1]

eps.shr <- str_split(eps.shr,pattern = " ")

eta.shr <- shr[grepl(shr[,1],pattern = "ETASHRINKSD"),1]

eta.shr <- str_split(eta.shr,pattern = " ")

shr.name <- c("etaCL","etaVC","etaKA","eps1","eps2")

shr <- c(eta.shr[[1]][c(2,3,4)],eps.shr[[1]][2:3])

shr <- signif(as.numeric(shr),3)

esti <- c("exp(theta1)","exp(theta2)","exp(theta3)",

"cov(etaCL,etaVC)",

"sigma1","sigma2")

length(esti)

desc <- c("CL (L/hr)","VC (L)","KA (1/hr)",

"Covariance of etaCL and etaVC",

"Proportional residual error (%)",

"Additional residual error (ng/mL)")

length(desc)

value <- out$par.rse[c(1:3,7,4,5)];length(value)

iiv <- out$par.rse[c(6,8,9,NA,NA,NA)];length(iiv)

shrinkage <- shr[c(1,2,3,NA,4:5)]

output <- data.frame(esti,desc,value,iiv,shrinkage)

write.csv(output,paste(DIRout,"Table . Base Model parameter.csv",sep = ""),

row.names = F, na = "-")

# Final model parameter ####

no.model <- "FL"

ext <- read.csv(paste(DIRmodel,"model",no.model,".ext",sep = ""),skip = 1,sep = "")

parameter <- t(ext[ext$ITERATION=="-1000000000",])

se <- t(ext[ext$ITERATION=="-1000000001",])

out <- data.frame(parameter=parameter[,1],se=se[,1])

out <- out[2:(nrow(out)-1),]

out <- out[out$parameter!=0,]

out <- out[out$parameter!=1,]

out$rse <- out$se/out$parameter*100

out$row <- 1:nrow(out)

out$parameter[c(1:3)] <- exp(out$parameter[c(1:3)])

out$parameter[c(8,10,11)] <- sqrt(exp(out$parameter[c(8,10,11)])-1)*100

out$parameter[c(6)] <- sqrt(out$parameter[c(6)])*100

out$parameter[c(7)] <- sqrt(out$parameter[c(7)])

out$rse[c(1:3)] <- sqrt(exp(out$se[c(1:3)]^2)-1)*100

out$rse[c(6:8,10,11)] <- out$rse[c(6:8,10,11)]/2

out$par.rse <- paste(signif(out$parameter,3)," (",signif(out$rse,3),")",sep = "")

shr <- read.csv(paste(DIRmodel,"model",no.model,".lst",sep = ""))

eps.shr <- shr[grepl(shr[,1],pattern = "EPSSHRINKSD"),1]

eps.shr <- str_split(eps.shr,pattern = " ")

eta.shr <- shr[grepl(shr[,1],pattern = "ETASHRINKSD"),1]

eta.shr <- str_split(eta.shr,pattern = " ")

shr.name <- c("etaCL","etaVC","etaKA","eps1","eps2")

shr <- c(eta.shr[[1]][c(2,3,4)],eps.shr[[1]][2:3])

shr <- signif(as.numeric(shr),3)

esti <- c("exp(theta1)","exp(theta2)","exp(theta3)","theta4","theta5",

"cov(etaCL,etaVC)",

"sigma1","sigma2")

length(esti)

desc <- c("CL (L/hr)","VC (L)","KA (1/hr)",

"DBIL on CL","WT on VC",

"Covariance of etaCL and etaVC",

"Proportional residual error (%)",

"Additional residual error (ng/mL)")

length(desc)

value <- out$par.rse[c(1:5,9,6,7)];length(value)

iiv <- out$par.rse[c(8,10,11,NA,NA,NA,NA,NA)];length(iiv)

shrinkage <- shr[c(1,2,3,NA,NA,NA,4:5)]

output <- data.frame(esti,desc,value,iiv,shrinkage)

write.csv(output,paste(DIRout,"Table . Final Model parameter.csv",sep = ""),

row.names = F, na = "-")

# bootstrap ####

no.model <- "FL"

ext <- read.csv(paste(DIRmodel,"model",no.model,".ext",sep = ""),skip = 1,sep = "")

parameter <- t(ext[ext$ITERATION=="-1000000000",])

se <- t(ext[ext$ITERATION=="-1000000001",])

out <- data.frame(parameter=parameter[,1],se=se[,1])

out <- out[2:(nrow(out)-1),]

out <- out[out$parameter!=0,]

out <- out[out$parameter!=1,]

out$up <- out$parameter+1.96*out$se

out$down <- out$parameter-1.96*out$se

out$NO <- 1:nrow(out)

out[c(1:3),c(1,3,4)] <- exp(out[c(1:3),c(1,3,4)])

out[c(8,10,11),c(1,3,4)] <- sqrt(exp(out[c(8,10,11),c(1,3,4)])-1)*100

out[c(6),c(1,3,4)] <- sqrt(out[c(6),c(1,3,4)])*100

out[c(7),c(1,3,4)] <- sqrt(out[c(7),c(1,3,4)])

out$est <- signif(out$parameter,3)

DIRbs <- paste(DIRmodel,"bs_modelBS/",sep = "")

bs <- read.csv(paste(DIRbs,"bootstrap_results.csv",sep = ""),skip = 25)

bs <- data.frame(t(bs[c(1,6,9),c(3:5,7,10,16,18:19,17,20:21)]))

colnames(bs) <- c("median","p2.5","p97.5")

bs$median <- as.numeric(bs$median)

bs$p2.5 <- as.numeric(bs$p2.5)

bs$p97.5 <- as.numeric(bs$p97.5)

bs[c(1:3),] <- exp(bs[c(1:3),])

bs[c(6:8),] <- sqrt(exp(bs[c(6:8),])-1)*100

bs[10,] <- sqrt(bs[10,])*100

bs[11,] <- sqrt(bs[11,])

bs$median <- signif(bs$median,3)

bs$p95 <- paste(signif(bs$p2.5,3),"~",signif(bs$p97.5,3))

out <- out[c(1:5,8,10:11,9,6,7),]

output <- cbind(bs,out)

nrow(output)

output$par <- c("exp(theta1)","exp(theta2)","exp(theta3)","theta4","theta5",

"omega1","omega2","omega3",

"cov(etaCL,etaVC)",

"eps1","eps2")

length(output$par)

output$desc <- c("CL (L/hr)","VC (L)","KA (1/hr)",

"DBIL on CL","WT on VC","IIV_CL","IIV_VC","IIV_KA",

"Covariance of etaCL and etaVC",

"Proportional residual error (%)","Additional residual error (ng/mL)")

output$bias <- signif((output$median/output$est-1)*100,3)

output$median <- signif(output$median,3)

output$ci95 <- paste(signif(output$down,3),"~",signif(output$up,3))

output0 <- output[,c("par","desc","est","ci95","median","p95","bias")]

colnames(output0) <- c("Parameter","Description","Final model estimates","Final model estimates 95%CI",

"Bootstrap median","Bootstrap 95% PI","Bootstrap bias (%)")

write.csv(output0,paste(DIRout,"Table . Bootstrap.csv",sep = ""),

row.names = F, na = "-")

# pairs and hist of ETAs ####

panel.cor <- function(x, y, digits = 2, prefix = "", cex.cor, ...)

{

usr <- par("usr"); on.exit(par(usr))

par(usr = c(0, 1, 0, 1))

r <- cor(x, y)

txt <- format(c(r, 0.123456789), digits = digits)[1]

txt <- paste0(prefix, txt)

if(missing(cex.cor)) cex.cor <- 0.8/strwidth(txt)

# text(0.5, 0.5, txt, cex = cex.cor * r)

text(0.5, 0.5, txt, cex = 1)

}

panel.hist <- function(x, ...)

{

usr <- par("usr"); on.exit(par(usr))

par(usr = c(usr[1:2], 0, 1.5) )

h <- hist(x, plot = FALSE,breaks=10)

breaks <- h$breaks; nB <- length(breaks)

y <- h$counts; y <- y/max(y)

rect(breaks[-nB], 0, breaks[-1], y, col = "lightblue", ...)

}

no.model <- "FL"

RES <- read.csv(paste(DIRmodel,"model",no.model,".RES",sep = ""),skip = 1,sep = "")

data <- RES[RES$MDV==0,]

base <- data[!duplicated(data$ID),]

X <- paste("ETA",c(1,2,3),sep = "")

LABELs <- paste("eta",c("CL","VC","KA"),sep = "")

pdf(paste(DIRout,"Figure . Pairwise scatterplot and histogram of etas of model",no.model,".pdf",sep = ""),width = 7.5,height = 6)

pairs(base[,X], lower.panel = panel.smooth, upper.panel = panel.cor,diag.panel = panel.hist,

gap=0, row1attop=FALSE,labels = LABELs)

dev.off()

# Individual PK parameter ####

no.model <- "FL"

RES <- read.csv(paste(DIRmodel,"model",no.model,".RES",sep = ""),skip = 1,sep = "")

data <- RES[RES$MDV==0,]

base <- data[!duplicated(data$ID),]

colnames(base)

write.csv(base[,c("ID","CL","V","KA")],

paste(DIRout,"Table . Individual PK parameter.csv",sep = ""),row.names = F)

# VPC ####

library(lattice)

library(xpose4)

DIRvpc <- "../pk/vpc_model24/"

pdf(paste(DIRout,"Figure . VPC-TSLD.pdf",sep = ""),height = 6,width = 7.5)

options(scipen=999)

xpose.VPC(vpc.info = paste(DIRvpc,"vpc_results.csv",sep = ""),

vpctab = paste(DIRvpc,"vpctab",sep = ""),

logy = F,main = "Visual Predictive Check",xlb= "Time Since Last Dose (hr)",ylb = "Prediction Corrected Concentration (ng/mL)",

PI.identify.outliers = F,PI.limits=c(0.05,0.95))

dev.off()
